# Comparison of microsatellite instability detection by immunohistochemistry and molecular techniques in colorectal and endometrial cancer

**DOI:** 10.1038/s41598-021-91974-x

**Published:** 2021-06-18

**Authors:** Franceska Dedeurwaerdere, Kathleen BM Claes, Jo Van Dorpe, Isabelle Rottiers, Joni Van der Meulen, Joke Breyne, Koen Swaerts, Geert Martens

**Affiliations:** 1 Department of Pathology, AZ Delta General Hospital, Roeselare, Belgium; 2 Center for Medical Genetics, Ghent University Hospital, Gent, Belgium; 3 Department of Pathology, Ghent University Hospital, Gent, Belgium; 4 Department of Laboratory Medicine, Department of Laboratory Medicine, AZ Delta General Hospital, AZ Delta General Hospital, Deltalaan 1, 8800 Roeselare, Belgium; 5 Department of Biomolecular Medicine, Ghent University, Gent, Belgium; 6 Cancer Research Institute Ghent (CRIG), Ghent University, Gent, Belgium; 7 Molecular Diagnostics, Ghent University Hospital, Gent, Belgium

**Keywords:** Cancer, Molecular biology, Biomarkers, Molecular medicine, Oncology

## Abstract

DNA mismatch repair deficiency (dMMR) testing is crucial for diagnosing Lynch syndrome and detection of microsatellite unstable (MSI) tumors eligible for immunotherapy. The aim of this study was to compare the relative diagnostic performance of three molecular MSI assays: polymerase chain reaction (PCR), MSI testing by Idylla and next-generation-sequencing (NGS) on 49 tumor samples (28 colorectal and 21 endometrial adenocarcinomas) versus immunohistochemistry (IHC). Discrepancies were investigated by *MLH1* methylation analysis and integrated with germline results if available. Overall, the molecular assays achieved equivalent diagnostic performance for MSI detection with area under the ROC curves (AUC) of respectively 0.91 for Idylla and PCR, and 0.93 for NGS. In colorectal cancers with tumor cell percentages ≥ 30% all three molecular assays achieved 100% sensitivity and specificity (AUC = 1) versus IHC. Also, in endometrial cancers, all three molecular assays showed equivalent diagnostic performance, albeit at a clearly lower sensitivity ranging from 58% for Idylla to 75% for NGS, corresponding to negative predictive values from 78 to 86%. PCR, Idylla and NGS show similar diagnostic performance for dMMR detection in colorectal and endometrial cancers. Molecular MSI analysis has lower sensitivity for dMMR detection in endometrial cancer indicating that combined use of both IHC and molecular methods is recommended.

Clinical Trial Number/IRB: B1172020000040, Ethical Committee, AZ Delta General Hospital.

## Introduction

Microsatellites are DNA elements composed of short repetitive motifs that are prone to misalignment and frameshift mutations during cell division. In healthy cells, the ensuing small indels or single-base mispairs are corrected by heterodimer enzyme complexes of the DNA mismatch repair (MMR) system encoded by the key MMR genes *MLH1*, *MSH2*, *PMS2* and *MSH6*^1^. dMMR results in the progressive accumulation of genetic mutations with each cell replication, potentially dysregulating many oncogenes or tumor suppressor genes. The molecular hallmark of dMMR is MSI (microsatellite instability), with expansions or contractions in the number of tandem repeats throughout the genome. This phenomenon is observed in a considerable proportion of colorectal, endometrial, gastric, pancreatic, brain, biliary tract, urinary tract and ovarian tumors^1^.

The MSI phenotype is most commonly caused by loss of MLH1 protein expression secondary to transcriptional suppression by *MLH1* gene promoter hypermethylation^2^. Alternatively, dMMR is observed in patients with Lynch syndrome, caused by autosomal dominant heterozygous germline variants in *MLH1*, *MSH2*, *PMS2*, *MSH6,* or a large deletion encompassing one or more exons of *EPCAM* and the promoter region of *MSH2*. These patients have an increased risk for colorectal, endometrial, gastric, pancreatic, brain, biliary tract, urinary tract and ovarian tumors^1,3^. In a proportion of Lynch syndrome like tumors without an identifiable pathogenic germline variant, double somatic variants are detected in a mismatch repair gene^4,5^.

As dMMR is caused by loss of function variants in the mismatch repair genes, immunohistochemistry (IHC) for MLH1, MSH2, MSH6 and PMS2 is used as pre-screening for Lynch syndrome and MMR status^1,6–10^. An alternative approach is to look for microsatellite instabilities by fragment length analysis of fluorescent PCR products of microsatellite loci^7,8,10,11^. Reflex testing for dMMR/MSI is considered standard clinical practice in the work-up of colorectal^12–15^, gastric^16^ and endometrial cancer^17,18^. In these tumor types, MSI status is an element needed for correct molecular classification. MSI status also influences therapeutic decisions: for instance, microsatellite unstable colorectal cancer is preferably not treated with 5-fluoro-uracil^19^ but may be eligible for treatment with immunotherapy^20,21^. The recognition of MSI as predictive biomarker for immunotherapy increased the clinical importance of adequate dMMR testing.

PCR-based microsatellite testing is widely used as standard technique to determine MSI status. More recently, MSI analysis by next-generation sequencing (NGS) was introduced^22–25^. Hereby relevant tandem repeat loci are included in targeted NGS panels and indel length distributions of aligned reads are analysed. Another recent option is the fully automated Idylla MSI assay developed by Biocartis, which measures MSI using seven repeat markers within 150 min per sample including DNA extraction^26,27^.

The aim of this study was to investigate if Idylla MSI assay and MSI detection by NGS using the mSINGS secondary analysis are diagnostically equivalent for determining MSI status in both colorectal and endometrial adenocarcinoma as compared to the widely used fluorescent PCR for 5 mononucleotide and 3 dinucleotide microsatellite loci. To this end, we performed a blinded head-to-head comparison of diagnostic performance of Idylla MSI assay, NGS and PCR using IHC for MLH1, MSH2, MSH6 and PMS2 as independent reference technique.

## Materials and methods

### Study specimens

**Figure 1:**
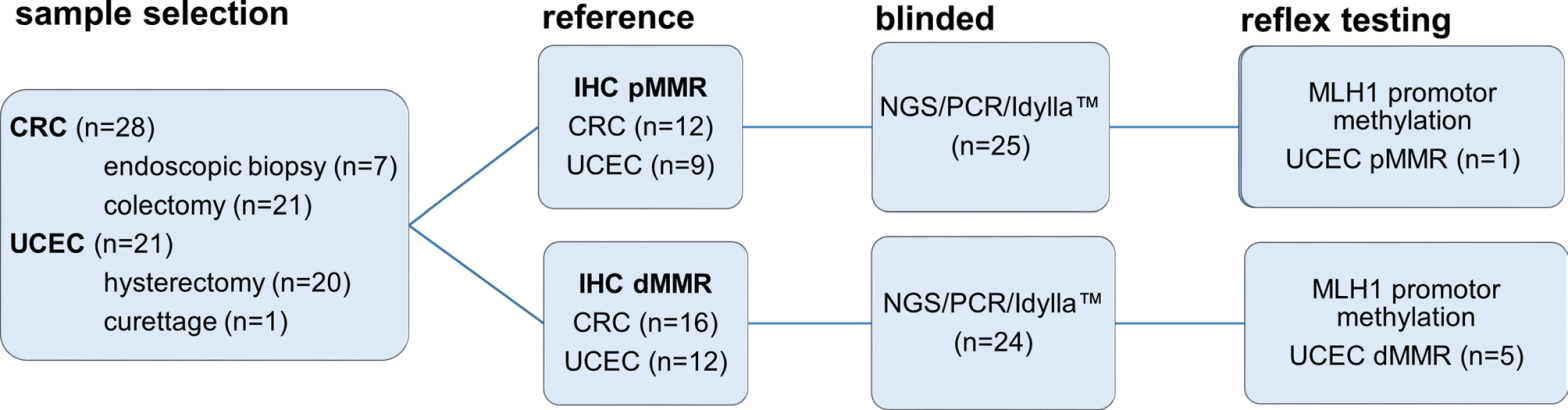
Flowchart of study design. Colorectal (CRC, n = 28) and uterine corpus endometrial cancers (UCEC, n = 21) were classified as DNA mismatch repair (MMR) deficient (dMMR) or proficient (pMMR) based on immunohistochemistry as reference technique (loss of expression of MLH1, PMS2, MSH2, MSH6) and then subjected to blinded analysis of MSI status by three molecular MSI assays. In selected cases, reflex testing was done for *MLH1* promoter methylation after unblinding of IHC and molecular test results.

Diagnostic performance was evaluated on 28 colorectal cancers and 21 endometrial cancer specimens selected from the archives of AZ Delta General Hospital’s pathology lab (Fig. 1, Supplementary Table S1). MMR status was determined by IHC for mismatch repair proteins MSH2, MSH6, PMS2 and MLH1 during the initial diagnostic work-up. Colorectal cancers included 16 MMR-deficient (dMMR) and 12 MMR- proficient (pMMR) adenocarcinoma and were obtained by endoscopy (n = 7) or colectomy (n = 21) from patients with a median age of 70 years (95%CI 65–77 years). Endometrial cancers included 12 dMMR and 9 pMMR endometrial adenocarcinomas obtained by hysterectomy (n = 20) or curettage (n = 1) from patients with a median age of 70 years (95% CI 65–74 years). Median tumor cell percentage as determined by hematoxylin and eosin (H&E) staining was 40% (range 20–75%). All selected cases underwent parallel molecular microsatellite instability (MSI) testing by fluorescent polymerase chain reaction (PCR) and the Idylla MSI assay (both at Ghent University Hospital) and Next-Generation Sequencing (NGS) at AZ Delta (Fig. 1). All three molecular assays and immunohistochemical scoring were performed strictly blinded from each other by separate teams. All specimens were obtained from patients as part of standard clinical and diagnostic care. The study was conducted in accordance with the Declaration of Helsinki. The study was approved by the AZ Delta Ethical Committee (Clinical Trial Number/IRB B1172020000040, study 20126, approved on November 23, 2020) with a waiver of informed consent since the study relied only on secondary use of biomaterials and data that were previously obtained as part of standard medical care.

### MSI testing by immunohistochemistry

Immunohistochemistry for MSH2, MSH6, PMS2 and MLH1 was performed on 5-µm thick sections of a representative formalin-fixed, paraffin-embedded (FFPE) tumor tissue block on a Ventana, Benchmark Ultra device (Ventana Medical Systems, Arizona, USA). Tumors were classified as mismatch repair deficient if no nuclear staining or nuclear staining in less than 10% of invasive tumor cells for 1 or several markers was seen in the presence of a positive internal control (inflammatory and stromal cells). Tumors with nuclear staining for all for markers in at least 10% of invasive tumor cells are considered to be pMMR. Detailed experimental protocols are provided in Supplementary Information.

### DNA extraction for PCR and NGS

DNA was extracted from 10-µm thick sections of the same FFPE tumor tissue blocks as used for immunohistochemistry, using the Cobas DNA Sample Preparation Kit (Roche, Basel, Switzerland) with macrodissection guided by hematoxylin eosin staining where needed, with elution in 10 mM Tris–HCl pH8.0.

### MSI testing by NGS

NGS was performed on an Illumina MiSeq device using a customized hybridization capture-based (NimbleGen SeqCap EZ HyperPlus, Roche) gene panel that included 15 microsatellite loci (Supplementary Table S2) exactly as described by Salipante et al.^23^. After read alignment against the human reference genome (hg19) with BWA (version 0.7.3a)^28^ and SAMtools (version 0.1.18)^29^, the mSINGS (version v3.6) open source python script (Python v.3.7, Matplotlib 3.3.4) was used to count and visualize the number of discrete indel length peaks (peak threshold > 5% of aligned reads) per locus to obtain a binary scoring for each locus as stable/unstable, where a locus is called unstable if a higher number of indel lenth peaks is measured as compared to a reference set of immunohistochemically MSS/pMMR colorectal cancers. In our analysis, only 10 of 15 original loci were diagnostically informative with area under the ROC (AUC) curve greater than 0.5 (*KDM6A*, *SMARCB1*, *GRIN2A*, *FLT1*, *CDK4*, *KTM2A*, *KIF5B*, *BCL2L11*, *MSH6* and *EML4*). The percentage of unstable loci per sample (mSINGS score) is then used for binary calling at sample level as MSI/MSS using > 30% (3/10) unstable loci as Youden index-optimized cutoff. Limit of detection of NGS-mSINGS is set at 30% tumor cells.meaning that in samples with tumor cell percentage below 30% a positive (MSI) result is considered valid but a negative (MSS) is reported as inconclusive.

### MSI testing by fluorescent PCR

Eight microsatellite loci were analysed, including five mononucleotide (*BAT-25*, *BAT-26*, *NR21*, *NR24* and *NR27*) and three dinucleotide markers (*D2S123*, *D17S250* and *D18S55*) selected based on published recommendations^11,30–33^. For interpretation purposes, microsatellite instability at ≥ 2 loci was defined as MSI-high, instability at a single locus was defined as MSI-low, and no instability at any of the loci tested was defined as MSS. Limit of detection is set at 30% tumor cells.

### MSI testing by Idylla

The Idylla MSI assay automates the entire process from FFPE DNA extraction to reporting of MSI status: DNA extracted from 10-µm thick FFPE sections is PCR amplified and scored by high-resolution melt analysis using a panel of 7 biomarkers (*ACVR2A*, *BTBD7*, *DID01*, *MRE11*, *RYR3*, *SEC31A* and *SULF*2)^34^. For adequate results a minimal tumor cell percentage of 20% and a minimum of 5/7 valid biomarker results is needed. The output sums up the MSI status for each biomarker (‘mutation detected’ or ‘no mutation detected’) and gives a conclusion based on the number of MSI positive biomarkers. A sample is considered Microsatellite Instability-High or MSI-H if it has ≥ 2 biomarkers with a 'mutation detected' biomarker call. The Idylla MSI assay was used under ‘for research use only’ label for endometrial cancers. The assay is CE-IVD certified only for use on FFPE colorectal cancer samples.

### Testing for *MLH1* promoter methylation status by PCR

The SALSA MS-MLPA kit ME011 (MRC-Holland, Amsterdam, The Netherlands) was used to evaluate if aberrant CpG island methylation in the promoter of *MLH1*^35,36^ exactly according to manufacturer’s instructions (details in the supplementary data S1 and S3).

### Statistical analysis

The diagnostic performance of the three molecular methods for detection of microsatellite instability was evaluated by calculating area under the receiver operating characteristics (ROC) curve (AUC) compared to IHC as reference test. Statistical differences between ROC curves and 95% confidence intervals were evaluated using the method of Delong et al.^37^ The authors did not account for multiple comparisons. Statistical analyses were performed using MedCalc (version 12.2.1, MedCalc Software, Mariakerke, Belgium, www.medcalc.org) and considered significant if P value was less than 0.05.

## Results

### Diagnostic performance of molecular panel-based testing in colorectal and uterine corpus endometrial cancers

**Figure 2:**
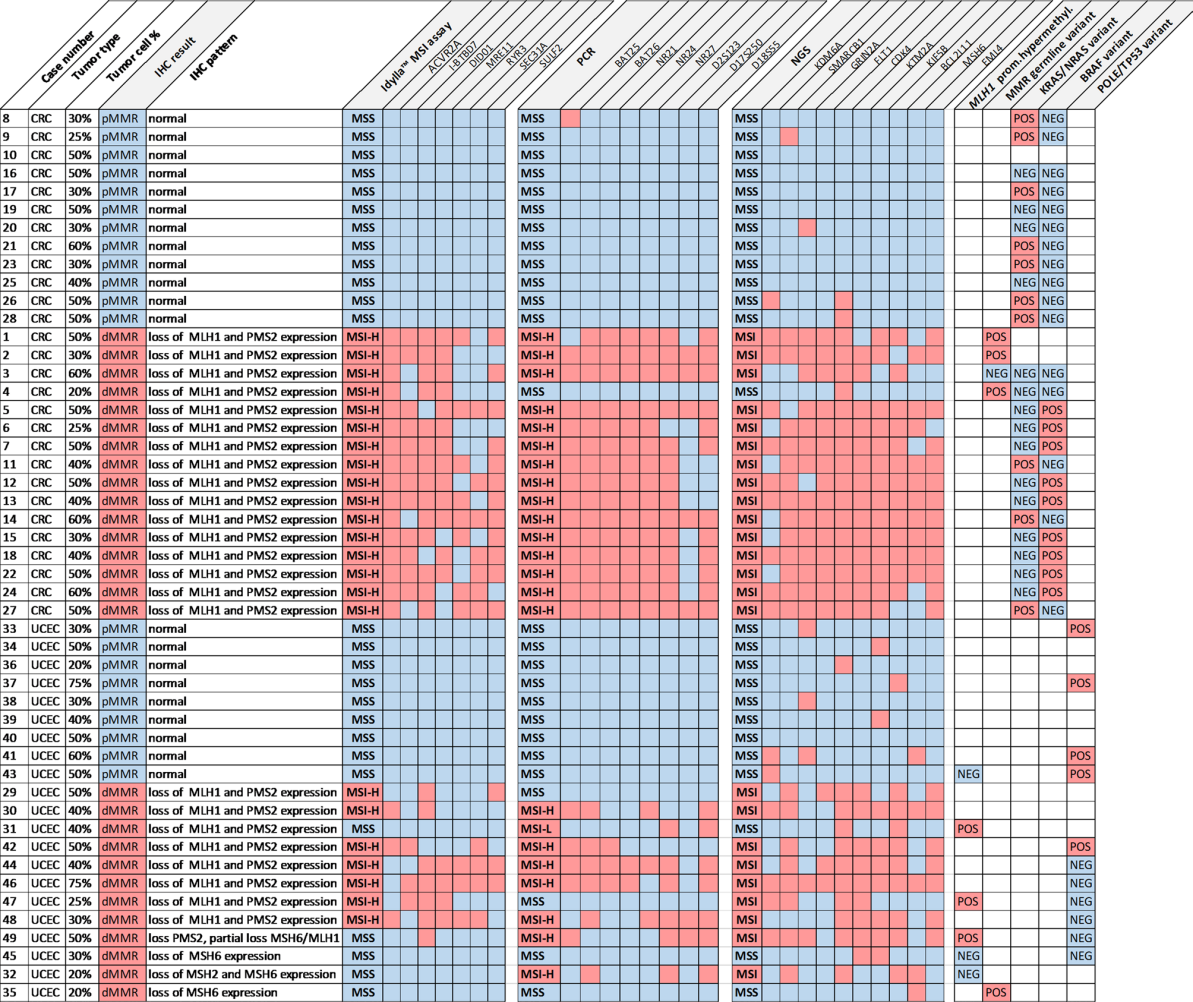
MSS/MSI calling at locus level and integrative panel-based result for Idylla MSI assay, PCR and NGS versus immunohistochemistry. Samples are grouped according to colorectal (CRC) or uterine corpus endometrial carcinoma (UCEC) tumor type and MLH1/PMS2/MSH2/MSH6 immunohistochemical expression pattern with from top to bottom: pMMR CRC (n = 12), dMMR CRC (n = 16), pMMR UCEC (n = 9) and dMMR UCEC (n = 12) samples with the indicated tumor cell percentage, IHC pattern and case number. From left to right the results are shown of MSI calling in the 7, 8 and 10 loci of the Idylla MSI test, PCR and NGS-respectively. For each molecular technique the overall integrative binary scoring as microsatellite stable (MSS, blue) or microsatellite unstable (MSI, MSI-L: MSI-low; MSI-H: MSI-high, red) is listed first, followed by the scoring of the individual loci. Right columns indicate the presence (red, POS: positive) or absence (blue, NEG: negative) of pathogenic variants in NRAS/KRAS and BRAF in CRC and *MLH1* promoter hypermethylation and (likely) pathogenic germline variants in MMR genes (MLH1, MSH2, MSH6, PMS2) in UCEC. Blank fields indicates parameter not assessed. Details on identified variants in supplementary Table S1.

Diagnostic performance of the three molecular assays was evaluated in 49 selected tumor samples (flowchart Fig. 1): colorectal (CRC, n = 28) and uterine corpus endometrial carcinoma (UCEC, n = 21) were classified as dMMR or pMMR using immunohistochemical detection of MLH1, PMS2, MSH6 and MLH1 expression as reference technique, and subjected to blinded analysis of MSI status by the three molecular techniques. Idylla MSI assay, PCR and NGS provide an integrative binary assessment of microsattelite instability based on the analysis of indel length distribution in respectively 7, 8 and 10 microsatellite loci (graphical overview in Fig. 2, raw data in Supplementary Tables S4, S5, S6).

Idylla MSI assay, PCR and NGS achieved similar diagnostic power in all tumor samples (n = 49) with statistically identical area under the receiver operating characteristics (ROC) curve (AUC) of 0.91 (95% CI 0.79–0.97), 0.91 (95% CI 0.79–0.97) and 0.93 (95% CI 0.82–0.98), respectively (Table 1). All three molecular assays achieved 100% specificity resulting in 100% positive predictive value (PPV). Sensitivities in all samples were also similar, ranging from 82 to 86%. All three molecular assays showed better diagnostic performance in CRC than in UCEC, but within each tumor type their diagnostic power as measured by AUC was equivalent.

**Table 1:** Diagnostic performance of three molecular MSI tests versus IHC as reference test in colorectal (CRC) and uterine corpus endometrial (UCEC) cancer.

All samples (n = 49)	AUC	95% CI	Sensitivity	95% CI	Specificity	95% CI	Accuracy	95% CI	PPV*	NPV*
Idylla MSI assay	0.91	0.79 to 0.97	82%	63 to 94%	100%	84 to 100%	97%	88 to 100%	100%	94%
PCR	0.91	0.79 to 0.97	82%	63 to 94%	100%	84 to 100%	97%	88 to 100%	100%	94%
NGS	0.93	0.82 to 0.98	86%	67 to 96%	100%	84 to 100%	98%	88 to 100%	100%	95%

UCEC (n = 21)	AUC	95% CI	Sensitivity	95% CI	Specificity	95% CI	Accuracy	95% CI	PPV	NPV*
Idylla MSI assay	0.79	0.56 to 0.94	58%	28 to 85%	100%	66 to 100%	94%	74 to 100%	100%	78%
PCR	0.83	0.61 to 0.96	67%	35 to 90%	100%	66 to 100%	95%	76 to 100%	100%	82%
NGS	0.88	0.66 to 0.98	75%	43 to 95%	100%	66 to 100%	96%	78 to 100%	100%	86%

CRC (n = 28)	AUC	95% CI	Sensitivity	95% CI	Specificity	95% CI	Accuracy	95% CI	PPV	NPV*
Idylla MSI assay	1.00	0.88 to 1.00	100%	79 to 100%	100%	74 to 100%	100%	88 to 100%	100%	100%
PCR	0.97	0.82 to 1.00	94%	70 to 100%	100%	74 to 100%	99%	86 to 100%	100%	99%
NGS	0.97	0.82 to 1.00	94%	70 to 100%	100%	74 to 100%	99%	86 to 100%	100%	99%

Overview of diagnostic performance expressed as area under the receiver operating characteristics (ROC) curve (AUC), sensitivity, specificity and accuracy with 95% confidence intervals of the three molecular assays in all samples (n = 49) or grouped according to colorectal (CRC, n = 28) or uterine corpus endometrial cancer (UCEC, n = 21) tumor type. The negative and positive predictive values (NPV, PPV) are calculated assuming a prevalence of microsatellite unstable tumors in real-world clinical practice of 15% in CRC, 40% in UCEC and 26% in all samples.

*AUC* area under the receiver operating characteristics (ROC) curve, *CI* confidence interval, *NPV and PPV* negative and positive predictive value

*NPV and PPV calculated assuming a typical prevalence of MSI status of 40% in UCEC, 15% in CRC and 26% in all samples.

In CRC, sensitivity was 100% (95% CI 79%-100%) for Idylla MSI assay. PCR and NGS were both falsely negative in the same CRC sample (case 4 in Fig. 2, detailed in Fig. 5) with a low percentage of tumor cells (20%) which is sufficient for the Idylla MSI assay but below the optimal tumor cell percentage of at least 30% tumor cells for confident calling by PCR and NGS. Assuming a prevalence of 15% microsatellite unstable tumors in CRC, these high sensitivities translate into excellent negative predictive values (NPV) of 99–100%.

In UCEC, specificity of molecular assays was also 100%. Sensitivity, however, was clearly lower ranging from 58% (95% CI 28–85%) for Idylla MSI assay, 67% (95% CI 35–90%) for PCR to 75% (95% CI 43–95%) for NGS (Table 1). In a typical clinical cohort of endometrial cancers with 40% prevalence of MSI^38^, this translates into NPV of 78% for Idylla MSI assay, 82% for PCR and 86% for NGS. When diagnostic performance was expressed versus the consensus result of all three molecular tests (Supplementary Table S7), NGS achieved the highest sensitivity (90%, 95% CI 55–100%) at 100% specificity in endometrial cancers, though not significantly higher than the sensitivity of PCR (80%, 95% CI 44–98%) or Idylla MSI assay (70%, 95% CI 35–93%).

### Diagnostic performance of individual loci versus panel-based approach in molecular assays

**Figure 3:**
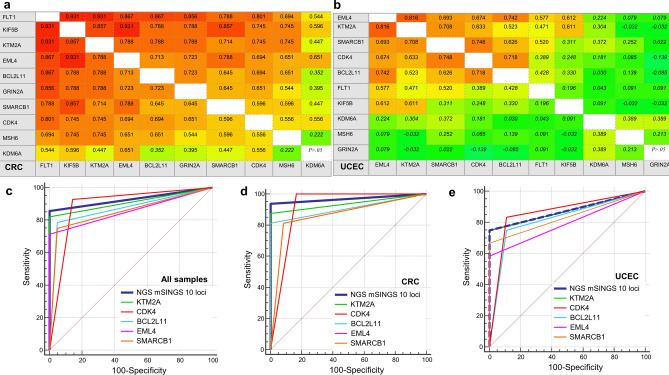
functional redundancy in diagnostic performance of individual loci and overall inferior performance of all loci in endometrial versus colorectal cancers. Figure 3 shows the results of the 10 loci in the NGS assay but similar data were obtained for PCR and Idylla MSI assay. Panel (**a**) and (**b**) show correlation tables with non-parametric Spearman rank correlation coefficients of AUC of individual microsatellite loci for detection of dMMR status versus IHC in CRC (**a**) and UCEC (**b**). Coefficients in italic font indicate non statistically significant correlation (*P* > 0.05). Coefficients colored according to the magnitude of the correlation. Loci with the highest inter-correlation also ranked among the highest AUC (Table 2). Panel c shows graphic plot of AUC of the integrated NGS result over 10 loci (thick blue line) versus the 5 loci with the highest individual AUC over all 49 samples (**c**). Panel d and e plot AUC in CRC (n = 28) and UCEC (n = 21) separately. Plots created by MedCalc (version 12.2.1, www.medcalc.org).

The panels of individual microsatellite loci used in panel-based molecular tests were initially selected and optimized for colorectal cancers associated with Lynch syndrome. Their inferior diagnostic performance in endometrial cancers might be explained by the reported tendency of some loci towards more frequent instability in specific tumor types, suggesting the existence of tumor type-associated instability patterns^39^. The panel of the Idylla MSI assay was designed to overcome this issue, by selecting loci shown to be unstable across various tumor types^34^. To investigate if specific individual loci show a preferential superior performance in the challenging UCEC tumors, we calculated the AUC of all individuall loci in the three molecular panel-based tests and compared it to the integrative binary result for the total panel. We found that the AUC of all individual loci in all three assays were systematically lower in UCEC versus CRC samples (Table 2 and graphically shown for NGS assay in Fig. 3c).

**Table 2:** Diagnostic performance of individual microsatellite loci within the panel-based assays in colorectal (CRC) and uterine corpus endometrial (UCEC) cancer.

Diagnostic performance for individual microsatellite loci in NGS assay											
	All samples					CRC			UCEC		
	AUC	95% CI		Sens (%)	Spec (%)	AUC	95% CI		AUC	95% CI	
KTM2A	0.911	0.794 to 0.973		82	100	0.938	0.777 to 0.994		0.875	0.658 to 0.977	
CDK4	0.893	0.771 to 0.963		93	86	0.917	0.749 to 0.987		0.861	0.641 to 0.971	
BCL2L11	0.869	0.742 to 0.948		79	95	0.906	0.735 to 0.983		0.819	0.592 to 0.951	
EML4	0.857	0.728 to 0.941	§	71	100	0.906	0.735 to 0.983		0.792	0.561 to 0.936	
SMARCB1	0.851	0.720 to 0.937	§	75	95	0.865	0.682 to 0.964		0.833	0.608 to 0.958	
KIF5B	0.827	0.693 to 0.920	§	75	90	0.938	0.777 to 0.994		0.681	0.444 to 0.864	
FLT1	0.821	0.686 to 0.916	§	64	100	0.969	0.823 to 0.999		0.625	0.390 to 0.823	
MSH6	0.726	0.580 to 0.844	§	50	95	0.813	0.621 to 0.934	§	0.611	0.377 to 0.813	
GRIN2A	0.690	0.542 to 0.815	§	57	81	0.896	0.722 to 0.979		0.583	0.351 to 0.791	
KDM6A	0.661	0.511 to 0.790	§	46	86	0.74	0.540 to 0.886	§	0.556	0.326 to 0.769	
NGS 10 loci	0.929	0.818 to 0.982		86	100	0.969	0.823 to 0.999		0.875	0.658 to 0.977	

Diagnostic performance for individual microsatellite loci in Biocartis Idylla MSI assay											
	All samples					CRC			UCEC		
	AUC	95% CI		Sens (%)	Spec (%)	AUC	95% CI		AUC	95% CI	
DID01	0.875	0.749 to 0.952		75	100	0.938	0.777 to 0.994		0.792	0.561 to 0.936	
ACVR2A	0.839	0.706 to 0.929	§	68	100	1	0.877 to 1.000		0.625	0.390 to 0.823	§
MRE11	0.804	0.665 to 0.903	§	61	100	0.906	0.735 to 0.983		0.667	0.430 to 0.854	
BTBD7	0.768	0.625 to 0.876	§	54	100	0.875	0.695 to 0.969	§	0.625	0.390 to 0.823	§
SULF2	0.768	0.625 to 0.876	§	54	100	0.875	0.695 to 0.969	§	0.625	0.390 to 0.823	§
SEC31A	0.696	0.549 to 0.820	§	39	100	0.719	0.518 to 0.871	§	0.667	0.430 to 0.854	
RYR3	0.696	0.549 to 0.820	§	39	100	0.75	0.551 to 0.893	§	0.625	0.390 to 0.823	§
Idylla 7 loci	0.911	0.794 to 0.973		82	100	1	0.877 to 1.000		0.792	0.561 to 0.936	

Diagnostic performance for individual microsatellite loci in PCR assay											
	All samples					CRC			UCEC		
	AUC	95% CI		Sens (%)	Spec (%)	AUC	95% CI		AUC	95% CI	
BAT26	0.865	0.734 to 0.947		75	100	0.964	0.808 to 0.999		0.75	0.516 to 0.910	
D2S123	0.846	0.711 to 0.935		71	100	0.929	0.756 to 0.992		0.75	0.516 to 0.910	
D18S55	0.827	0.689 to 0.922	§	68	100	0.857	0.665 to 0.962		0.792	0.561 to 0.936	
NR21	0.827	0.689 to 0.922	§	67	100	0.967	0.816 to 0.999		0.636	0.395 to 0.836	§
NR27	0.827	0.689 to 0.922	§	64	100	0.967	0.816 to 0.999		0.636	0.395 to 0.836	§
BAT25	0.822	0.683 to 0.918	§	70	100	0.923	0.748 to 0.990		0.708	0.472 to 0.883	
NR24	0.808	0.666 to 0.908	§	61	100	0.967	0.816 to 0.999		0.591	0.352 to 0.802	§
D17S250	0.635	0.481 to 0.770	§	27	100	0.679	0.468 to 0.847	§	0.583	0.351 to 0.791	§
PCR 8 loci	0.904	0.782 to 0.970		82	100	0.964	0.808 to 0.999		0.833	0.608 to 0.958	

Area under the ROC curve (AUC) with 95% confidence intervals (CI) for prediction of MMR status using IHC as reference test were calculated for the 10, 7 and 8 individual microsatellite loci in the NGS, Idylla MSI and PCR test and statistically compared to the integrative result for each of the three molecular panel-tests. This was done for all samples (n = 49) with calculation of sensitivity (Sens%) and specificity (Spec%), and separately for both tumor types. § indicates significantly different (P < 0.05) AUC for the individual locus as compared to the integrative result of the corresponding panel test.

*AUC* area under the receiver operating characteristics (ROC) curve, *95% CI* 95% confidence interval, *Sens* sensitivity, *Spec* specificity. § Indicates significantly different AUC (*P* < 0.05) of individual microsatellite locus versus integrated result over 10, 7 and 8 loci for NGS, Idylla MSI assay and PCR respectively.

This analysis also indicates that individual loci within the panel-based tests provide largely redundant diagnostic information and are strongly correlated. For instance, in the NGS test, the AUC of the top 3 best performing loci (*KTM2A*, *CDK4* and *BCL2L11*) are statistically similar to the integrative result over the 10 loci, both for CRC and UCEC samples (Table 2). In one multiple logistic regression model to predict dMMR/pMMR IHC status, only *KIF5B* (*P* = 0.0137) and *CDK4* (*P* = 0.0005) were retained as independent predictors (not shown). The diagnostic redundancy is also illustrated by the high degree of correlation of the loci with the highest AUC both in CRC (Fig. 3a) and UCEC (Fig. 3b). Similar correlations were observed for loci embedded in the PCR and Idylla MSI assay (Supplementary Figure 1).

### Discrepant IHC and molecular MSI calling in samples with loss of MSH6 expression

7 of 12 (58%) of endometrical cancers scored as dMMR by IHC were falsely called MSS/pMMR by at least one the three molecular assays. In 4 of these 7 cases this was associated with loss of MSH6 expression, isolated (n = 2) or combined (n = 2) with loss of other MMR proteins (Fig. 2). The 2 cases with isolated loss of MSH6 protein expression (Fig. 2, case 35 and 45) were the only cases that were called MSS by all three molecular methods; in case 35, the MMR deficient phenotype was additionally confirmed by a likely pathogenic germline variant in the *MSH6* gene (c.3744_3773del, p.(His1248_Ser1257del)). In 3 of 7 cases, the MMR deficient phenotype was additionally confirmed by *MLH1* promoter hypermethylation.

### Selected illustrative cases

**Figure 4:**
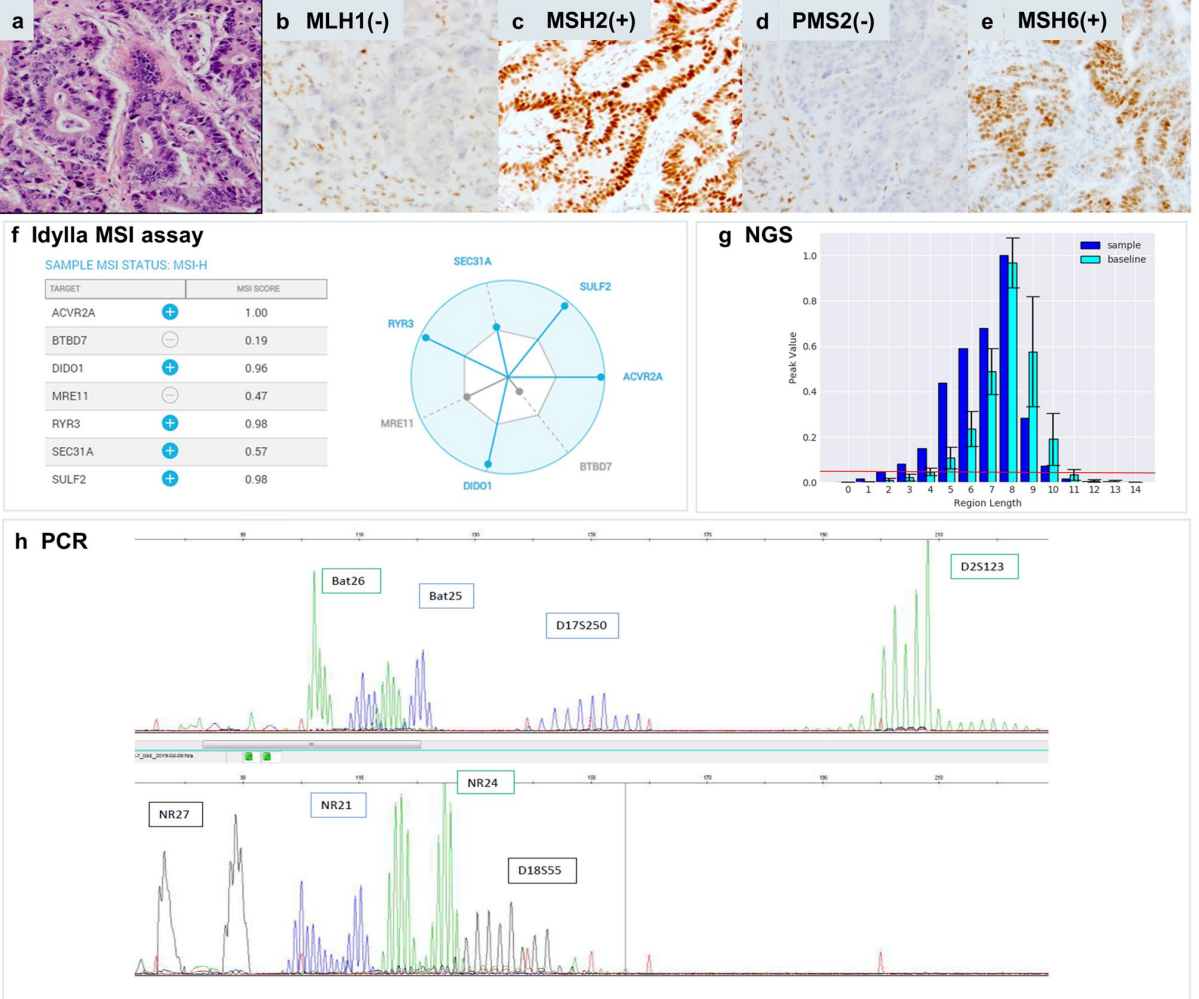
Colorectal dMMR/MSI tumor (case 27) with concordant results between IHC and all three molecular methods. Hematoxylin eosin stain (**a**) of the tumor with classic morphology of colorectal adenocarcinoma with combined nuclear loss of expression of MLH1 (**b**) and PMS2 (**d**) and preserved nuclear staining for MSH2 (**c**) and MSH6 (**e**). Idylla MSI assay (**f**) indicates 5/7 loci MSI resulting in a global MSI-high score (Idylla Explore software v.2.5.1294). Illustrative indel distribution plot of the FLT1 locus with clearly higher numbers of integrated peaks. An indel length peak is integrated by mSINGS and counted when the fraction of aligned reads per indel length is higher than 5% of total reads for the entire locus (peak value, > 0.05, threshold for peak integration indicated by red line (plot using Python v.3.7 and Matplotlib v.3.3.4). Here the number of integrated peaks on the FLT1 locus in the test sample (dark blue, 8 peaks above threshold) is higher than number of peaks in the trained baseline (light blue, 6 peaks) thus binary calling this locus as MSI. Representative fragment length distributions as measured by PCR (GeneMapper v. 4.0 analysis software, Applied Biosystems). (**g**) and manually interpreted as MSI by a trained observer. (**h**) PCR results for 8 microsatellite markers clearly show a wide range of alleles for all loci.

Case 27 (Figs. 2, 4) is a CRC sample with 50% tumor cells with combined loss of MLH1 and PMS2 expression and concordant true positive results in all three molecular assays. For NGS (Fig. 4g) a typical shift in indel distribution is shown, with widening of the distribution and clearly increased number of indel lenghts peaks in the tumor sample as compared to a MSS control set. Similarly, a wide distribution of alleles for all microsatellites is clear from the peak patterns obtained by PCR (Fig. 4h).

**Figure 5:**
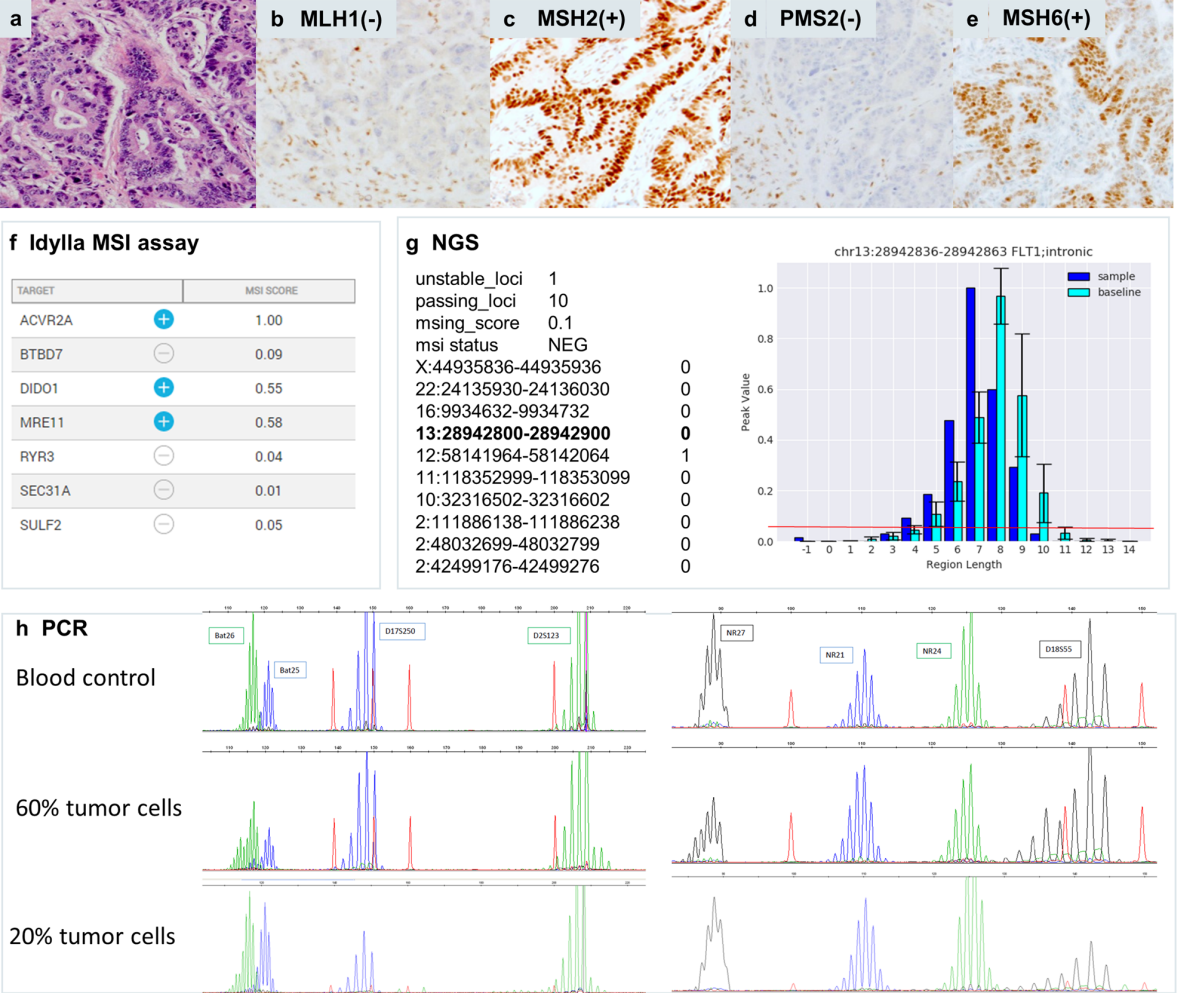
Colorectal dMMR/MSI tumor (case 4) with concordant results between IHC and Idylla MSI assay but falsely negative by PCR and NGS due to low tumor cell percentage. Hematoxylin eosin stain (**a**) of the tumor with classic morphology of colorectal adenocarcinoma with combined nuclear loss of expression of MLH1 (**b**) and PMS2 (**d**) and preserved nuclear staining for MSH2 (**c**) and MSH6 (**e**). A sample with 20% tumor cells was extracted and analyzed by the three molecular methods. Idylla MSI assay (**f**) indicated 3/7 loci MSI resulting in an integrative MSI-high score (Idylla Explore software v.2.5.1294). By NGS (**g**) only 1 of 10 loci was called unstable (Python v.3.7 and Matplotlib 3.3.4 software), resulting in an integrative falsely negative MSS result. Illustrative indel distribution plot of the FLT1 locus: both in the tumor (dark bue bar) and in baseline (light blue bars) 6 indel peaks were integrated above the threshold (red line, > 0.05 peak value or 5% of total reads for this locus). A clear shift towards shorter indel lenghts was visible in the indel distribution of the tumor sample but this did not result in a higher number of integrated peaks versus baseline, leading to calling as MSS/0. The PCR (h) on the 20% tumor cell sample was also falsely negative (GeneMapper v. 4.0 analysis software, Applied Biosystems). Repeat analysis of case 4 in a biopsy with 60% tumor cells did result in true positive MSI calling, with widening of indel distribution as compared to normal tissue (blood sample) of this same patient, panel h right part).

Case 4 (Figs. 2, 5) was the only dMMR CRC sample in our series that was missed by both PCR (0/8 loci MSI) and NGS (1/10 loci MSI) likely due to a low tumor cell percentage (20%) and correctly classified by Idylla MSI assay (3/7 loci MSI). This sample was obtained from an individual with Lynch syndrome due to a germline variant in the *MLH1* gene (c.882C > T; r.791_884del; (p.His264Leufs*2)). On retesting another FFPE tumor block with higher tumor cell percentage (60%), MSI was confirmed by PCR (NGS not repeated). This case highlights a possible limitation in the default parametrization of the mSINGS script. mSINGS counts the number of discrete peaks in the indel distribution, whereby a peak is only recognized when it holds at least 5% of total reads for that locus. A locus is scored MSI/1 when the total number of peaks in the distributions is higher than the total number of peaks in a baseline control set of MSS samples^23^. In case 4, the indel distribution at the *FLT1* locus on chromosome 13 is clearly left-shifted towards shorter indel lenghts but since total number of peaks is not altered, the locus is called MSS/0 (Fig. 4g).

**Figure 6:**
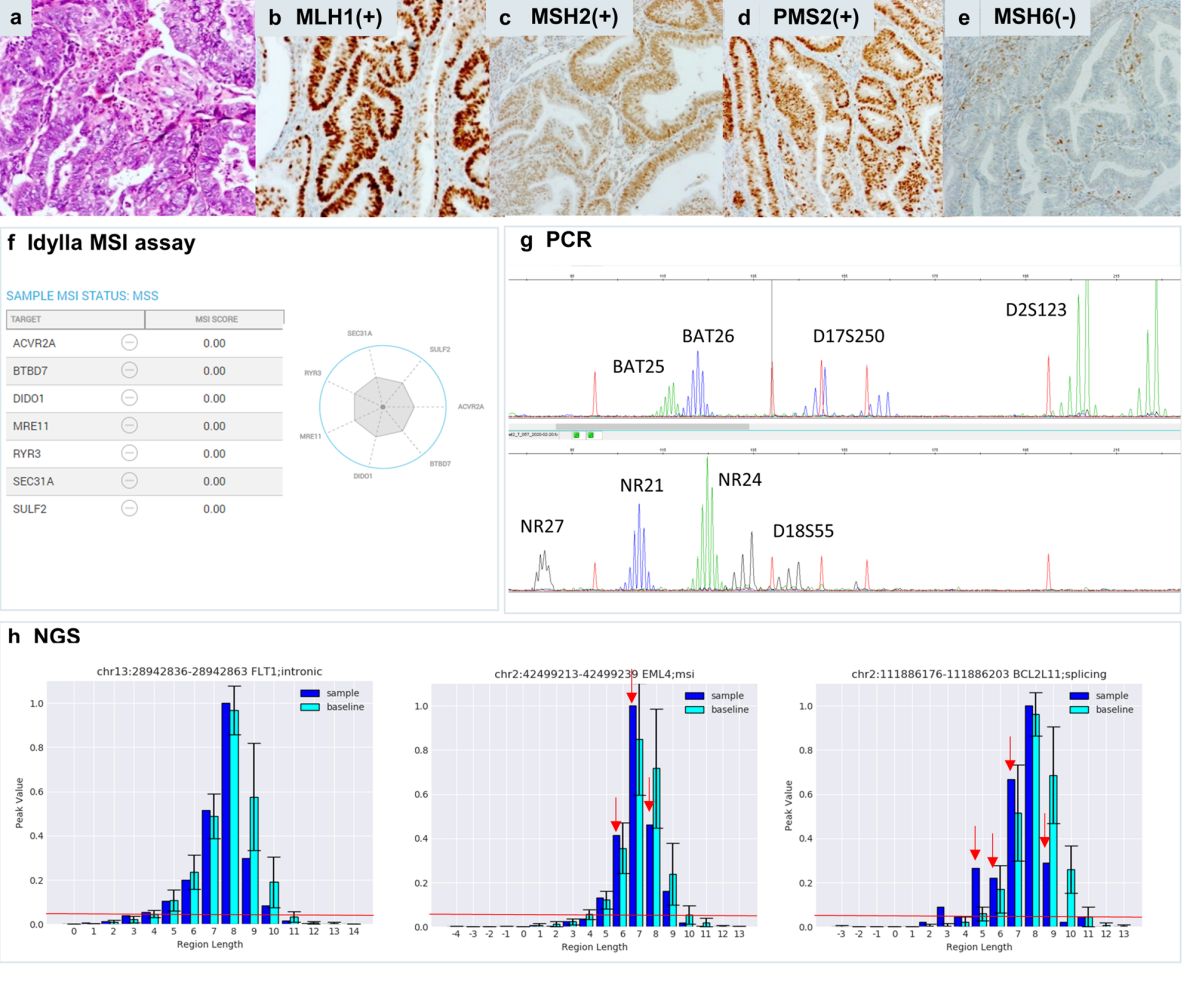
Endometrial dMMR/MSI tumor (case 45) with isolated loss of MSH6 expression, falsely negative by Idylla MSI assay, PCR and NGS. Hematoxylin eosin stain (**a**) of the tumor with classic morphology of endometrial adenocarcinoma (UCEC) with isolated loss of nuclear MSH6 (**e**) and preserved nuclear expression of MLH1 (**b**), MSH2 (**c**) and PMS2 (**d**). Idylla MSI assay (Idylla Explore software v.2.5.1294) (**f**) and PCR (GeneMapper v. 4.0 analysis software, Applied Biosystems). (**g**) called respectively 0/7 loci and 0/8 loci as MSS, resulting in MSS integrative result (Fig. 2). NGS (Python v.3.7 and Matplotlib 3.3.4 software) called 2/10 loci as unstable (Fig. 2) resulting in an overall MSS/0 score. Illustrative indel distribution plot of the FLT1, EML4 and BCL2L11 loci (**h**, from left to right) indicated identical number of integrated peaks (6/5/6 for respectively FLT1/ EML4/BCL2L11 loci) in tumor sample (dark blue bars) versus baseline (light blue bars) resulting in calling these loci as negative by the default mSINGS script. However, for the EML4 and BCL2L11 loci, the overall indel distribution did shift towards shorter indel lenghts as indicated by the red arrows, suggesting the presence of molecular alterations not recognized by the current parametrization of the script.

Case 45 (Figs. 2, 6) was an UCEC sample with 30% tumor cells and isolated loss of MSH6 by IHC. Idylla MSI assay (0/7 loci MSI), PCR (0/8 loci MSI) and NGS (2/10 loci MSI) were all negative. Here again, the indel distribution plots of NGS indicated minimal shifts in the indel distribution towards shorter read lenghts (e.g. in the *EML4* and *BCL2L11* loci, Fig. 6h) but without increased number of mSINGS-integrated discrete indel lenghts peaks, resulting in calling these loci as MSS/0.

## Discussion

The seminal study by Le et al.^20^ firmly established MSI as predictive biomarker for PD-1 blockade in dMMR tumors. Since the approval of pembrolizumab anti-PD-1 immunotherapy by the Food and Drug Administration in 2017 for the treatment of unresectable or metastatic, MSI-high/dMMR tumors, irrespective of the site of organ and histological subtype and irrespective of PDL-1 testing, detection of dMMR/MSI is considered a crucial tool for determining the therapy for many cancers. Therefore, good accessibility to accurate MSI testing should be guaranteed.

Immunohistochemical staining for MLH1/PMS2/MSH2/MSH6 and MSI analysis by fluorescent PCR with fragment length analysis are generally considered equivalent in diagnostic performance. Generally, there is a good concordance between both techniques. Recent ESMO consensus recommendations^40^ indicate IHC for the four key MMR proteins as the first test of choice and molecular analysis of the dMMR phenotype as mandatory confirmation if IHC is doubtful. Here we performed a head-to-head comparison of the classical IHC and PCR-based MSI methods with two alternative molecular methods, which provide automated operator-independent data analysis: NGS with indel length distribution analysis using the previously published mSINGS script and the fully automated Idylla MSI assay, respectively using a panel of 10 and 7 microsatellite regions. Our study indicates that NGS-mSINGS and Idylla MSI assay show a similar diagnostic performance as fluorescent PCR. Both in colorectal and endometrial cancers, all three molecular methods achieved 100% specificity, resulting in 100% positive predictive power, on samples with tumor cell percentages ranging from 20 to 75%, representative for a real-world clinical setting. In colorectal cancers, sensitivity and specificity were 100% for samples with a tumor cell percentage above 30%. Our results are in agreement with other recent reports on the concordance in colorectal cancers between the Idylla MSI assay and IHC/PCR^26,41–44^. Also in endometrial cancer, all molecular methods in our study achieved comparable sensitivity—albeit lower than the sensitivity observed in colorectal cancers. In previous studies on endometrial cancers, an acceptable concordance above 90% was found between IHC for MLH1/PMS2/MSH2/MSH6 and MSI analysis by fluorescent PCR^45–47^. Some authors consider IHC as the preferred method^48^, whereas others advize combining IHC with PCR^49^ and MLH1 promoter methylation testing^50^. Several studies warn for false negative results when screening for MSI with PCR only, particularly in case of MSH6 loss^48,49^. Others reported cases of tumors being pMMR by IHC and MSI (mostly MSI-L) by PCR^51^. Consequently, there is to date no universally accepted preference for one technique over the other and their combined use appears optimal to achieve maximal sensitivity. Each technique has its pros and its cons, as summarized in Table 3. Immunohistochemistry is rapid, widely available, inexpensive, gives information on which MMR gene is involved and can be used on FFPE biopsy samples with low tumor cell percentage. The stains are usually readily interpretable. However, false negative results occur due to fixation artefacts or unawareness of unusual staining patterns. False positive staining may occur in case of amino acid substitutions leading to loss of function with preserved immunoreactive protein expression^52^. Fluoresencent PCR was performed with a panel consisting of three dinucleotide microsatellite markers (D5S346, D2S123, D17S250) and five poly-A mononucleotide repeats (BAT-25, BAT-26, NR-21, NR-24 and NR-27)^40^. This is recommended because of superior sensitivity and specificity compared to the Bethesda- pentaplex panel with only two mononucleotide (BAT-25 and BAT-26) and the same dinucleotide markers . PCR is inexpensive but requires skilled analysts for interpretation of variations in fragment length distribution. For challenging cases, results may be operator-dependent and therefore, the technique is less amenable to automatic interpretation. The Idylla MSI assay is fast, does not require batching of samples, is fully automated (from sample extraction to data interpretation) and operator independent. However, it requires a dedicated instrument, has a relatively high cost per sample and provides no flexibility in terms of MSI panel design. It thus appears an optimal solution for labs with relatively low number of MSI analyses and limited experience. NGS is expensive, has relatively long turnaround times, requires in-house development and validation of bioinformatic pipelines and is generally not cost-effective as standalone test. However, for labs with sequencing capacity and for tumour types that are already sequenced as part of standard care, inclusion of a microsatellite panel is cost-effective. Moreover, NGS offers a high flexibility in terms of panel design with the possibility of developing tumour type-specific panels. Bioinformatic analysis of indel distrubtions requires strong validations, but with an established pipeline the analysis is o operator-independent and easily automatable. With this approach implementation of MSI analysis is cost-effective for all solid tumors undergoing sequencing as standard of care to identify actionable gene variants.

**Table 3:** Pros and cons of IHC versus molecular methods.

Characteristic	IHC	PCR	NGS	Idylla
**Cost/sample**				
(1) NGS required by guidelines	Low	Low	Low	High
(2) Stand-alone MSI testing	Low	Low	Very high	High
Turnaround time (days)	1–2	1–2	5–10	0.2
Information on MMR driver gene	Yes	No	No	No
Accessibility	High	Intermediate	Low	Intermediate
Minimally required tumor cell percentage	1%	30%	30%	20%
Operator dependence	Intermediate	Intermediate	Low	Low
Normal tissue as internal control	No	Difficult cases	No	No
Integration in standard workflow	Standard	Standalone test	Possible	Standalone test
MSI locus panel flexibility	Low	High	High	Low
CE-IVD/FDA	Yes	Yes	Variable	Yes
Other	–	–	–	Dedicated instrument

The table lists some pros and cons of IHC and the three molecular MSI tests. Generally, for tumor types that undergo default NGS analysis, the total runcost of NGS is very low once the investment in bioinformatic scripts and MSI integration in panel design are made and overall flexibility of MSI panel design and data analysis is very high. Idylla MSI assay is interesting for labs with low sample number and limited operator experience but requires a dedicated separate analyser and the closed systems limits analysis of locus-specific indel distribution patterns. PCR is inexpensive and currently considered as reference test along IHC but its dependence on experienced operators is higher.

A specific strength of our study is the head-to-head comparison on the same sample set, with four independent laboratories performing a blinded analysis with an individual technique, allowing a direct comparison. Furthermore, to resolve discrepancies, *MLH1* promoterhyper methylation by MS-MLPA was applied. This resolved discrepancyies in 3/6 (endometrial) cases—in favour of IHC. In addition, results of germline testing were available for some cases, further improving correct integration of the results obtained by the different techniques. A limitation of our study is the fact that it has a possible selection bias as IHC was used as reference method. Around 6% of cancers with PCR-confirmed MSI show preserved MLH1/PMS2/MSH2/MSH6 expression by IHC^53^.

Endometrial cancers are known to display minimal microsatellite shifts (one to three nucleotide repeat shifts in unstable locus) more frequently than colorectal cancers^54,55^. Our data are in line with previous reports that cancers with *MSH6* germline variants often display low or absent MSI^1,56^. This is explained by the fact that the MSH2-MSH6 heterodimer repairs single base-pair mismatches and dinucleotide insertion-deletion loops while the MSH2-MSH3 heterodimers specializes for larger insertion-deletion loops of 2–13 nucleotides. A recent study on 15 endometrial cancers concluded 100% sensitivity and specificity for the Idylla MSI system and pentaplex PCR-based assay; somewhat lower values were obtained for their targeted NGS approach^41^. However, IHC was doubtful for MSH6 in one of the endometrial tumors and the authors concluded pMMR as the molecular techniques showed concordance. However, case 35 (Fig. 2) in our study demonstrates that, despite concordance of the three molecular techniques, IHC correctly indicated loss of MSH6 expression since a germline pathogenic *MSH6* variant was demonstrated in this patient.

A genome-wide analysis of 200,000 microsatellite loci across 18 tumor types indicated that some microsatellite loci are more likely to be unstable in specific tumor types, suggesting that definition of tumor type-specific MSI panels might harbor increased analytical sensitivity^39^. However, for the 25 loci analyzed here in the aggregated results of Idylla MSI assay (7 loci), PCR (8 loci) and NGS (10 loci), we could not identify a single locus that was more likely to be unstable in endometrial than colorectal cancer. Further research is needed to investigate if a novel combination of the loci with highest AUC in endometrial cancers, selected from the panels of Idylla MSI assay, PCR and NGS, might further boost diagnostic performance for endometrial carcinoma.

Besides optimization of the studied loci, diagnostic performance in endometrial cancer might also be improved by improved parametrization of indel distribution analysis, in particular to account for the minimal microsatellite shifts. This might prove challenging for manually interpreted PCR data and for the fully automated Idylla MSI assay. NGS offers more flexibility here. As illustrated by the cases presented in Figs. 5 and 6, adaptations to the mSINGS script are needed, to not only detect increased numbers of discrete indel length peaks, but also to detect overall shifts in median indel length and skewing of its distribution. The relatively flexible panel design of NGS and its automated data analysis therefore appear technically most fit to exploit the potential of larger tumor-specific panels, and the prognostic power of the quantification of MSI burden in combination with simultaneous quantification of overall tumor mutation burden. Since most dMMR/MSI-prone cancers (colorectal, endometrial, pancreatic, ovarian) today are already sequenced to find actionable gene variants, NGS has the potential to become the method of choice for all tumor types, including rare tumor types not belonging to the spectrum of Lynch syndrome with low MSI prevalence, in line with a recent ESMO expert consensus recommendation^40^.

In conclusion, our study shows that Idylla MSI assay and NGS with mSINGS indel length distribution analysis achieve equivalent diagnostic performance as fluorescent PCR with a set of mono- and dinucleotide microsatellite markers. Sensitivity of all molecular techniques is higher in colorectal than in endometrial cancers. Patients with endometrial cancer found to be dMMR by IHC should be referred for *MLH1* gene promoter hypermethylation (in case of MLH1/PMS2 loss) and/or germline testing regardless of results of MSI testing by molecular methods. Our data support the standard combined use of IHC and a molecular MSI test to achieve maximally sensitive detection of tumors with DNA mismatch repair deficiency. Particularly for endometrial tumors, molecular analysis alone is currently insufficient. Awareness of this finding is important in order not to misclassify MSI/possible Lynch syndrome cases.

## Supplementary Information

Supplementary Information.
