# Supplementary material for: Comparison of microsatellite instability detection by immunohistochemistry and molecular techniques in colorectal and endometrial cancer
Source: Sci Rep. 2021 Jun 18;11:12880. doi: 10.1038/s41598-021-91974-x (PMC8213758; doi:10.1038/s41598-021-91974-x)
Supplement: Supplementary file 1 — Supplementary Information. [file 41598_2021_91974_MOESM1_ESM.docx]

**Supplementary information**

**Comparison of microsatellite instability detection by immunohistochemistry and molecular techniques in colorectal and endometrial cancer**

**Franceska Dedeurwaerdere** ^1,+^**, Kathleen BM Claes** ^2, 5, 7,+^**, Jo Van Dorpe** ^3,7^**, Isabelle Rottiers** ^3^**, Joni Van der Meulen ^2,7,8^, Joke Breyne** ^4^**, Koen Swaerts** ^4^**, Geert Martens** ^4,5,6^

^1^Department of Pathology, AZ Delta General Hospital, Roeselare, Belgium; ^2^Center for Medical Genetics, Ghent University Hospital, Gent, Belgium; ^3^Department of Pathology, Ghent University Hospital, Gent, Belgium; ^4^Department of Laboratory Medicine, AZ Delta General Hospital, Roeselare, Belgium, ^5^Department of Biomolecular Medicine, Ghent University, Gent, Belgium, ^6^VUB Metabolomics Group, Brussels Free University, Brussels, Belgium; ^7^Cancer Research Institute Ghent (CRIG), Ghent University, Gent, Belgium; ^8^Molecular Diagnostics, Ghent University Hospital, Gent, Belgium

**Corresponding author**: Geert A. Martens, Department of Laboratory Medicine, AZ Delta General Hospital, Deltalaan 1, 8800 Roeselare, Belgium. Phone: +32 51 233834 Email:geert.martens@azdelta.be, ORCID ID: 0000-0003-1208-6289

**Supplementary Methods: detailed description of Idylla™ MSI assay, NGS and fluorescent PCR**

**MSI testing by immunohistochemistry** Immunohistochemistry for MSH2, MSH6, PMS2 and MLH1 was performed on 5-µm thick sections of a representative formalin-fixed, paraffin-embedded (FFPE) tumor tissue block at the time of diagnosis. The stains were done on a Ventana, Benchmark Ultra device (Ventana Medical Systems, Arizona, USA). After incubation at 36°C for 32 minutes for MLH1 and MSH6, 12 minutes for MSH2 and for 48 minues for PMS2, deparaffinization and heat induced epitope retrieval with the Ventana Ultra CC1 buffer for 92 (PMS2), 64 (MLH1 and MSH6) or 32 (MSH2) minutes at maximum 98°C, the slides were stained with monoclonal antibodies for MSH2 (clone G219-1129, Ventana, Ready-to-Use), MSH6 (clone EP49, DAKO, dilution 1/50), PMS2 (A16-4, Ventana, Ready-to-use) and MLH1 (clone ES05, DAKO, dilution 1/50). For visualisation the OptiView DAB IHC Detection Kit (Ventana) was used for all 4 antibodies. Sections with positive and negative control tissues were added on each tumor slide. Tumors were classified as mismatch repair deficient if no nuclear staining or nuclear staining in less than 10% of invasive tumor cells for 1 or several markers was seen in the presence of a positive internal control (inflammatory and stromal cells). Tumors with nuclear staining for all for markers in at least 10% of invasive tumor cells are considered to be pMMR.

**DNA extraction for PCR and NGS** FFPE tumour tissues were analysed by a pathologist for neoplastic tumour cell content. If applicable, tumour-rich regions of interest were identified and marked on H&E stained slides and tissue material was macrodissected from unstained slides using H&E as a guide. DNA was extracted from 10-µm thick sections of the same FFPE tumor tissue blocks as used for immunohistochemistry, using the Cobas DNA Sample Preparation Kit (Roche, Basel, Switzerland) according to manufacturer’s protocol, with elution in 10 mM Tris-HCl pH8.0. DNA concentration was measured using a fluorometric method based on binding of double-stranded DNA (dsDNA)-selective fluorescent dyes (dsDNA) (Qubit 3.0 Fluorometer/Life Technologies).

**MSI testing by NGS** NGS was performed using a customized hybridization capture-based gene panel (NimbleGen SeqCap EZ HyperPlus, Roche) sequenced on an Illumina™ MiSeq device. The Seq Cap panel design included 15 microsatellite loci (Supplementary Table S2) for secondary analysis by the mSINGS script exactly as described by Salipante et al^1^. Read alignment against the human reference genome (hg19) was performed using BWA (version 0.7.3a)^2^ and SAMtools (version 0.1.18)^3^. mSINGS (version v3.6) is an open source python script that uses VARSCAN (version 2.3.7)^4^ and SAMTOOLS (version 0.1.18)^3^  to count the number of discrete indel length peaks (peak threshold >5% of aligned reads) within reads aligned to the predefined loci and then applies binary scoring for each locus as stable/unstable, where a locus is called unstable if a higher number of indel lenth peaks is measured as compared to a reference set of immunohistochemical MSS/pMMR (microsatellite stable/proficient mismatch repair) colorectal cancers. In our analysis, 10 of 15 original loci were retained in the analysis (*KDM6A*, *SMARCB1*, *GRIN2A*, *FLT1*, *CDK4*, *KTM2A*, *KIF5B*, *BCL2L11*, *MSH6* and *EML4*). Five loci were discarded since they displayed no variation at all in indel length distribution in our NGS chemistry/design (*ATM* and *PTPRD*), insufficient depth of coverage (*NF1*) or minimal variation with lack of diagnostic power (AUC < 0.6 for *TGFBR2* and *PBRM1*). The percentage of unstable loci per sample (mSINGS score) is then used for binary calling as MSI/MSS using > 30% (3/10) as Youden index-optimized cutoff with optimal area under the ROC (AUC) curve. Limit of detection of NGS-mSINGS is set at 30% tumor cells. Average dept of coverage per locus is 630 reads (range 312-943) with minimally 30 reads required for confident MSI calling.

**MSI testing by fluorescent PCR** Fluorescent PCRs were performed on the same DNA extract used for NGS. Eight microsatellite loci were analyzed, including five mononucleotide (*BAT-25*, *BAT-26*, *NR21*, *NR24* and *NR27*) and three dinucleotide markers (*D2S123*, *D17S250* and *D18S55*). These were selected based on previously published recommendations^5-9^. The primers were fluorescently labeled and PCR products were analyzed by capillary electrophoresis (CE) using ABI 3730XL Genetic Analyzer (Thermo Fisher Scientific, MA, USA). For interpretation purposes, microsatellite instability at ≥ 2 loci was defined as MSI-high, instability at a single locus was defined as MSI-low, and no instability at any of the loci tested was defined as MSS. Limit of detection is set at 30% tumor cells.

**MSI testing by Idylla™** The Idylla™ MSI assay was performed on 10-µm thick sections of the same FFPE tumor tissue blocks as used for testing for immunohistochemistry, NGS and PCR. The test liberates nucleic acids that are PCR amplified and scored by high-resolution melt analysis. The test automates the entire process from FFPE sample preparation to reporting of MSI status using the Biocartis Idylla™ system. MSI status is determined based on a panel of 7 biomarkers (*ACVR2A*, *BTBD7*, *DID01*, *MRE11*, *RYR3*, *SEC31A* and *SULF*2)^10^ . For adequate results a minimal tumor cell percentage of 20% and a minimum of 5/7 valid biomarker results is needed. The output sums up the MSI status for each biomarker (‘mutation detected’ or ‘no mutation detected’) and gives a conclusion based on the number of MSI positive biomarkers. A sample is considered Microsatellite Instability-High or MSI-H if it has ≥ 2 biomarkers with a 'mutation detected' biomarker call. The Idylla™ MSI assay was used under ‘for research use only’ label for endometrial cancers. The assay is CE-IVD certified only for use on FFPE colorectal cancer samples.

**Supplementary Table S1: overview of study cohort with sample characteristics and mismatch repair protein immunohistochemistry**

**Supplementary Table S2: genomic coordinates of microsatellite markers used for MSI detection by NGS**

The table list all 15 loci proposed by Salipante et al. ^1^. As explained in Materials and Methods section, only 10 of 15 loci (right column, YES) were retained in the analysis (KDM6A, SMARCB1, GRIN2A, FLT1, CDK4, KTM2A, KIF5B, BCL2L11, MSH6, EML4). 5 loci were discarded (right columns, NO) since they displayed no variation at all in indel length distribution in our NGS chemistry/design (ATM, PTPRD), insufficient depth of coverage (NF1) or minimal varation with lack of diagnostic power (AUC < 0.6 for TGFBR2 and PBRM1).

| gene | description | chromosome | start position | stop position | Included in mSINGS 10 loci |
| --- | --- | --- | --- | --- | --- |
| ATM | (T)13 | chr11 | 108188267 | 108188279 | NO |
| BCL2L11 | (T)18 | chr2 | 111886181 | 111886198 | YES |
| CDK4 | (A)14 | chr12 | 58142006 | 58142019 | YES |
| EML4 | (A)19 | chr2 | 424999218 | 42499236 | YES |
| FLT1 | (T)18 | chr13 | 28942841 | 2894258 | YES |
| GRIN2A | (A)20 | chr16 | 9934671 | 9934690 | YES |
| KDM6A | (T)13 | chrX | 4493878 | 4493890 | YES |
| KIF5B | (T)18 | chr10 | 32316542 | 32316559 | YES |
| KMT2A | (T)16 | chr11 | 118353038 | 118353053 | YES |
| MSH6 | (T)13 | chr2 | 48032741 | 48032753 | YES |
| NF1 | (T)16 | chr17 | 29508820 | 29508835 | NO |
| PBRM1 | (T)20 | chr3 | 526215887 | 52621606 | NO |
| PTPRD | (A)12 | chr9 | 8341281 | 8341292 | NO |
| SMARCB1 | (T)17 | chr22 | 24135968 | 24135985 | YES |
| TGFBR2 | (A)10 | chr3 | 30691872 | 30691881 | NO |

**Supplementary Data S3: protocol for MLH1 promotor methylation**

The MS-MLPA method is based on probes that recognize specific sequences in DNA that contains a restriction site for a methylation-sensitive HhaI enzyme. The target regions for the MLH1 gene silenced by promoter hypermethylation appear to be −248 to −178 (Deng C-region) and −9 to +15 (Deng D-region) (refs1+2). For this reason, methylation of the 202 nt and 172 nt probes are the most important determinant for mRNA expression.

MS-MLPA assays were performed as described by the manufacturer: in each reaction we used 200 ng of DNA (5 μl at 40 ng/μl). Similar to a conventional MLPA assay, genomic DNA is first denatured and subsequently cooled down to 25°C, followed by the addition of MS-MLPA probes and a 16-hour hybridization step. MS-MLPA assay is then split into two tubes; one tube is processed as a standard MLPA reaction: ligation of hybridized probe oligonucleotides followed by PCR amplification. The other tube of the MLPA hybridization reaction is incubated with the methylation-sensitive HhaI endonuclease. PCR was performed as described by the manufacturer, although we added double DNA–probe mix amount (10 μl) in each PCR reaction to improve results. Then, PCR fragments were separated and quantified by electrophoresis on an ABI 310 capillary analyzer (Applied Biosystems, Foster City, CA).

Methylation status for a tumor sample was calculated using GeneMapper v. 4.0 analysis software (Applied Biosystems). Peak height parameter is proportional to the amount of PCR product generated. To calculate the methylation ratio, each peak height from HhaI-digested tumor DNA was divided by its corresponding peak height from the undigested tumor DNA. To compensate for differences in PCR efficiency of the individual samples, each peak height (digested and undigested) was normalized dividing each probe amplification product by the average value of the 11 control probes without a HhaI enzyme site.[^21^](https://www.ncbi.nlm.nih.gov/pmc/articles/PMC2893635/#bib21)

The mean of MLH1 probe 3 and 4 corresponding to regions C and D, respectively, in MLH1 promoter were considered to calculate the methylation ratio. The dichotomization threshold to distinguish methylated versus non methylated samples was established at 15% based on a previous study associated with gene silencing.

**Supplementary Table S4: results of Idylla™ MSI test**

|  | **Idylla™ MSI Assay** | **ACVR2A** | **BTBD7** | **DID01** | **MRE11** | **RYR3** | **SEC31A** | **SULF2** |
| --- | --- | --- | --- | --- | --- | --- | --- | --- |
| **1** | MSI-H | mutant | mutant | mutant | mutant | mutant | wild type | mutant |
| **2** | MSI-H | mutant | mutant | mutant | mutant | wild type | wild type | wild type |
| **3** | MSI-H | mutant | wild type | mutant | mutant | wild type | wild type | mutant |
| **4** | MSI-H | mutant | wild type | mutant | mutant | wild type | wild type | wild type |
| **5** | MSI-H | mutant | mutant | wild type | mutant | mutant | mutant | mutant |
| **6** | MSI-H | mutant | mutant | mutant | mutant | wild type | wild type | wild type |
| **7** | MSI-H | mutant | mutant | mutant | mutant | wild type | wild type | mutant |
| **8** | MSS | wild type | wild type | wild type | wild type | wild type | wild type | wild type |
| **9** | MSS | wild type | wild type | wild type | wild type | wild type | wild type | wild type |
| **10** | MSS | wild type | wild type | wild type | wild type | wild type | wild type | wild type |
| **11** | MSI-H | mutant | mutant | mutant | mutant | mutant | wild type | mutant |
| **12** | MSI-H | mutant | mutant | mutant | mutant | wild type | mutant | mutant |
| **13** | MSI-H | mutant | mutant | mutant | mutant | mutant | wild type | mutant |
| **14** | MSI-H | mutant | wild type | mutant | mutant | mutant | mutant | mutant |
| **15** | MSI-H | mutant | mutant | mutant | wild type | mutant | wild type | mutant |
| **16** | MSS | wild type | wild type | wild type | wild type | wild type | wild type | wild type |
| **17** | MSS | wild type | wild type | wild type | wild type | wild type | wild type | wild type |
| **18** | MSI-H | mutant | mutant | wild type | mutant | wild type | mutant | mutant |
| **19** | MSS | wild type | wild type | wild type | wild type | wild type | wild type | wild type |
| **20** | MSS | wild type | wild type | wild type | wild type | wild type | wild type | wild type |
| **21** | MSS | wild type | wild type | wild type | wild type | wild type | wild type | wild type |
| **22** | MSI-H | mutant | mutant | mutant | mutant | wild type | mutant | mutant |
| **23** | MSS | wild type | wild type | wild type | wild type | wild type | wild type | wild type |
| **24** | MSI-H | mutant | mutant | mutant | wild type | mutant | mutant | wild type |
| **25** | MSS | wild type | wild type | wild type | wild type | wild type | wild type | wild type |
| **26** | MSS | wild type | wild type | wild type | wild type | wild type | wild type | wild type |
| **27** | MSI-H | mutant | wild type | mutant | wild type | mutant | mutant | mutant |
| **28** | MSS | wild type | wild type | wild type | wild type | wild type | wild type | wild type |
| **29** | MSI-H | wild type | wild type | mutant | wild type | wild type | wild type | mutant |
| **30** | MSI-H | mutant | wild type | mutant | wild type | wild type | wild type | wild type |
| **31** | MSS | wild type | wild type | wild type | wild type | wild type | wild type | wild type |
| **32** | MSS | wild type | wild type | wild type | wild type | wild type | wild type | wild type |
| **33** | MSS | wild type | wild type | wild type | wild type | wild type | wild type | wild type |
| **34** | MSS | wild type | wild type | wild type | wild type | wild type | wild type | wild type |
| **35** | MSS | wild type | wild type | wild type | wild type | wild type | wild type | wild type |
| **36** | MSS | wild type | wild type | wild type | wild type | wild type | wild type | wild type |
| **37** | MSS | wild type | wild type | wild type | wild type | wild type | wild type | wild type |
| **38** | MSS | wild type | wild type | wild type | wild type | wild type | wild type | wild type |
| **39** | MSS | wild type | wild type | wild type | wild type | wild type | wild type | wild type |
| **40** | MSS | wild type | wild type | wild type | wild type | wild type | wild type | wild type |
| **41** | MSS | wild type | wild type | wild type | wild type | wild type | wild type | wild type |
| **42** | MSI-H | mutant | mutant | wild type | wild type | wild type | mutant | wild type |
| **43** | MSS | wild type | wild type | wild type | wild type | wild type | wild type | wild type |
| **44** | MSI-H | wild type | wild type | mutant | mutant | mutant | mutant | mutant |
| **45** | MSS | wild type | wild type | wild type | wild type | wild type | wild type | wild type |
| **46** | MSI-H | wild type | mutant | mutant | mutant | mutant | mutant | mutant |
| **47** | MSI-H | wild type | mutant | mutant | mutant | wild type | wild type | wild type |
| **48** | MSI-H | mutant | wild type | mutant | mutant | mutant | mutant | wild type |
| **49** | MSS | wild type | wild type | mutant | wild type | wild type | wild type | wild type |

**Supplementary Table S5: results of MSI by PCR; i: microsatellite unstable locus; i?: possibly unstable locus, s: stable locus; 0: no PCR amplification; b: broader peak.** For binary calling at locus level, ‘s’, ‘i?’and ‘0’ were scored as 0 (microsatellite stable) and only loci scores as clearly microsatellite instable (‘I’) as 1. The integrative result of PCR includes three categories: microsatellite stable (MSS) and microsatellite instable – Low (MSI-L) and – high (MSI-H). For binary calling at integrative panel level, both MSI-H and MSI-L were considered ‘1/microsatellite instable).

|  | **PCR result** | **BAT25** | **BAT26** | **NR21** | **NR24** | **NR27** | **D2S123** | **D17S250** | **D18S55** |
| --- | --- | --- | --- | --- | --- | --- | --- | --- | --- |
| **1** | MSI-H | 0 | i | i | i | i | i | 0 | i |
| **2** | MSI-H | i | i | i | i | i | i | i | i |
| **3** | MSI-H | i | i | i | i | i | i | i | i |
| **4** | MSS | s | s | s | s | s | s | s | s |
| **5** | MSI-H | i | i | i | i | i | i | i | i |
| **6** | MSI-H | i | i | i | i | i | s | s | i |
| **7** | MSI-H | i | i | i | i | i | i | 0 | i |
| **8** | MSS | i | s | s | s | s | s | s | s |
| **9** | MSS | s | s | s | s | s | s | s | s |
| **10** | MSS | s | s | s | s | s | s | s | s |
| **11** | MSI-H | i | i | i | i | i | i | s | s |
| **12** | MSI-H | i | i | i | i | i | i | i? | i? |
| **13** | MSI-H | i | i | i | i | i | i | i? | i? |
| **14** | MSI-H | i | i | i | i | i | i | i | i |
| **15** | MSI-H | i | i | i | i | i | i | s | i |
| **16** | MSS | s | s | s | s | s | s | s | s |
| **17** | MSS | s | s | s | s | s | s | s | s |
| **18** | MSI-H | i | i | i | i | i | i | s | i |
| **19** | MSS | s | s | s | s | s | i? | s | s |
| **20** | MSS | s | s | s | s | s | s | s | s |
| **21** | MSS | s | s | s | s | s | s | s | s |
| **22** | MSI-H | i | i | i | i | i | i | s | i |
| **23** | MSS | s | s | s | s | s | s | s | s |
| **24** | MSI-H | i | i | i | i | i | i | s | i |
| **25** | MSS | s | s | s | s | s | s | s | s |
| **26** | MSS | s | s | s | s | s | s | s | s |
| **27** | MSI-H | i | i | i | i | i | i | i | i |
| **28** | MSS | s | s | s | s | s | s | s | s |
| **29** | MSS | s | s | s | s | s | s | s | s |
| **30** | MSI-H | i | i | s | s | i | s | s | i |
| **31** | MSI-L | s | i? | s | s | s | i | s | i |
| **32** | MSI-H | s | i | s | s | s | i | s | i |
| **33** | MSS | s | s | s | s | s | s | s | s |
| **34** | MSS | s | s | s | s | s | s | s | s |
| **35** | MSS | s | s | s | s | s | s | s | s |
| **36** | MSS | s | s | s | s | s | s | s | s |
| **37** | MSS | s | s | s | s | s | s | s | s |
| **38** | MSS | s | s | s | s | s | s | s | s |
| **39** | MSS | s | s | s | s | s | s | s | s |
| **40** | MSS | s | s | s | s | s | s | s | s |
| **41** | MSS | s | s | s | s | s | s | s | s |
| **42** | MSI-H | i | i | i | s | s | s | s | s |
| **43** | MSS | s | s | s | s | s | s | s | s |
| **44** | MSI-H | i | i | i | i | i | i | s | i |
| **45** | MSS | s | s | s | s | s | s | s | s |
| **46** | MSI-H | i | i | i | i | s | i | s | i |
| **47** | MSS | b | b | b | b | s | s | s | s |
| **48** | MSI-H | s | i | s | s | i | i | i | i |
| **49** | MSI-H | i | i? | b | b | s | i | i | i |

**Supplementary Table S6: results of MSI testing by NGS.** 1: unstable locus; 0: stable locus

|  | **mSINGSscore** | **result** | **KDM6A** | **SMARCB1** | **GRIN2A** | **FLT1** | **CDK4** | **KTM2A** | **KIF5B** | **BCL2L11** | **MSH6** | **EML4** |
| --- | --- | --- | --- | --- | --- | --- | --- | --- | --- | --- | --- | --- |
| **1** | 0,80 | POS | 1 | 1 | 1 | 1 | 1 | 0 | 1 | 1 | 0 | 1 |
| **2** | 0,90 | POS | 1 | 1 | 1 | 1 | 1 | 1 | 1 | 0 | 1 | 1 |
| **3** | 0,50 | POS | 0 | 0 | 1 | 1 | 1 | 1 | 0 | 1 | 0 | 0 |
| **4** | 0,10 | NEG | 0 | 0 | 0 | 0 | 1 | 0 | 0 | 0 | 0 | 0 |
| **5** | 0,90 | POS | 1 | 0 | 1 | 1 | 1 | 1 | 1 | 1 | 1 | 1 |
| **6** | 0,80 | POS | 0 | 1 | 1 | 1 | 1 | 1 | 1 | 1 | 1 | 0 |
| **7** | 0,90 | POS | 1 | 1 | 1 | 1 | 1 | 1 | 1 | 1 | 0 | 1 |
| **8** | 0,00 | NEG | 0 | 0 | 0 | 0 | 0 | 0 | 0 | 0 | 0 | 0 |
| **9** | 0,10 | NEG | 0 | 1 | 0 | 0 | 0 | 0 | 0 | 0 | 0 | 0 |
| **10** | 0,00 | NEG | 0 | 0 | 0 | 0 | 0 | 0 | 0 | 0 | 0 | 0 |
| **11** | 0,90 | POS | 0 | 1 | 1 | 1 | 1 | 1 | 1 | 1 | 1 | 1 |
| **12** | 0,90 | POS | 1 | 1 | 0 | 1 | 1 | 1 | 1 | 1 | 1 | 1 |
| **13** | 1,00 | POS | 1 | 1 | 1 | 1 | 1 | 1 | 1 | 1 | 1 | 1 |
| **14** | 0,90 | POS | 0 | 1 | 1 | 1 | 1 | 1 | 1 | 1 | 1 | 1 |
| **15** | 0,90 | POS | 0 | 1 | 1 | 1 | 1 | 1 | 1 | 1 | 1 | 1 |
| **16** | 0,00 | NEG | 0 | 0 | 0 | 0 | 0 | 0 | 0 | 0 | 0 | 0 |
| **17** | 0,00 | NEG | 0 | 0 | 0 | 0 | 0 | 0 | 0 | 0 | 0 | 0 |
| **18** | 1,00 | POS | 1 | 1 | 1 | 1 | 1 | 1 | 1 | 1 | 1 | 1 |
| **19** | 0,00 | NEG | 0 | 0 | 0 | 0 | 0 | 0 | 0 | 0 | 0 | 0 |
| **20** | 0,10 | NEG | 0 | 0 | 1 | 0 | 0 | 0 | 0 | 0 | 0 | 0 |
| **21** | 0,00 | NEG | 0 | 0 | 0 | 0 | 0 | 0 | 0 | 0 | 0 | 0 |
| **22** | 0,90 | POS | 0 | 1 | 1 | 1 | 1 | 1 | 1 | 1 | 1 | 1 |
| **23** | 0,00 | NEG | 0 | 0 | 0 | 0 | 0 | 0 | 0 | 0 | 0 | 0 |
| **24** | 0,90 | POS | 1 | 1 | 1 | 1 | 1 | 1 | 1 | 1 | 0 | 1 |
| **25** | 0,00 | NEG | 0 | 0 | 0 | 0 | 0 | 0 | 0 | 0 | 0 | 0 |
| **26** | 0,20 | NEG | 1 | 0 | 0 | 0 | 1 | 0 | 0 | 0 | 0 | 0 |
| **27** | 0,80 | POS | 1 | 1 | 1 | 1 | 1 | 1 | 1 | 0 | 0 | 1 |
| **28** | 0,10 | NEG | 0 | 0 | 0 | 0 | 1 | 0 | 0 | 0 | 0 | 0 |
| **29** | 0,60 | POS | 0 | 1 | 0 | 1 | 1 | 1 | 0 | 1 | 0 | 1 |
| **30** | 0,80 | POS | 1 | 1 | 0 | 0 | 1 | 1 | 1 | 1 | 1 | 1 |
| **31** | 0,20 | NEG | 0 | 0 | 0 | 0 | 1 | 0 | 0 | 1 | 0 | 0 |
| **32** | 0,40 | POS | 0 | 1 | 0 | 0 | 1 | 0 | 0 | 1 | 1 | 0 |
| **33** | 0,10 | NEG | 0 | 0 | 1 | 0 | 0 | 0 | 0 | 0 | 0 | 0 |
| **34** | 0,10 | NEG | 0 | 0 | 0 | 0 | 0 | 0 | 1 | 0 | 0 | 0 |
| **35** | 0,10 | NEG | 0 | 0 | 0 | 0 | 0 | 0 | 0 | 0 | 1 | 0 |
| **36** | 0,10 | NEG | 0 | 0 | 0 | 0 | 1 | 0 | 0 | 0 | 0 | 0 |
| **37** | 0,10 | NEG | 0 | 0 | 0 | 0 | 0 | 0 | 0 | 1 | 0 | 0 |
| **38** | 0,10 | NEG | 0 | 0 | 1 | 0 | 0 | 0 | 0 | 0 | 0 | 0 |
| **39** | 0,10 | NEG | 0 | 0 | 0 | 0 | 0 | 0 | 1 | 0 | 0 | 0 |
| **40** | 0,00 | NEG | 0 | 0 | 0 | 0 | 0 | 0 | 0 | 0 | 0 | 0 |
| **41** | 0,30 | NEG | 1 | 0 | 1 | 0 | 0 | 0 | 0 | 0 | 1 | 0 |
| **42** | 0,60 | POS | 0 | 1 | 0 | 0 | 1 | 1 | 1 | 1 | 0 | 1 |
| **43** | 0,10 | NEG | 1 | 0 | 0 | 0 | 0 | 0 | 0 | 0 | 0 | 0 |
| **44** | 0,70 | POS | 0 | 1 | 0 | 1 | 1 | 1 | 1 | 1 | 0 | 1 |
| **45** | 0,20 | NEG | 0 | 0 | 0 | 0 | 0 | 1 | 1 | 0 | 0 | 0 |
| **46** | 1,00 | POS | 1 | 1 | 1 | 1 | 1 | 1 | 1 | 1 | 1 | 1 |
| **47** | 0,40 | POS | 1 | 1 | 0 | 0 | 1 | 1 | 0 | 0 | 0 | 0 |
| **48** | 0,50 | POS | 0 | 0 | 0 | 0 | 1 | 1 | 1 | 1 | 0 | 1 |
| **49** | 0,80 | POS | 1 | 1 | 1 | 0 | 1 | 1 | 1 | 1 | 0 | 1 |

**Supplementary table S7: comparative diagnostic performance expressed as consensus value of the three molecular tests.** A sample was considered as true positive MSI when 2 or 3 of 3 molecular assays indicated MSI status. This led to reclassification of case 31 (IHC-MSI+ and MSI-Low by PCR but MSS by NGS and Idylla. For case 4 the consensus molecular result was negative but this was overruled because tumor cell percentage (20%) was below the limit of detection for NGS and Idylla, explaining their false negative results.

**Supplementary Figure 1** Legend to supplementary figure 1: Correlation table of individual microsatellite loci for detection of microsatellite instability for (a) Idylla™ MSI test; (b) PCR and (c) NGS for all samples (n=49). Table lists non-parametric Spearman rank correlation coefficients and are colored according to the magnitude of the correlation. Plot created by MedCalc (version 12.2.1, www.medcalc.org).


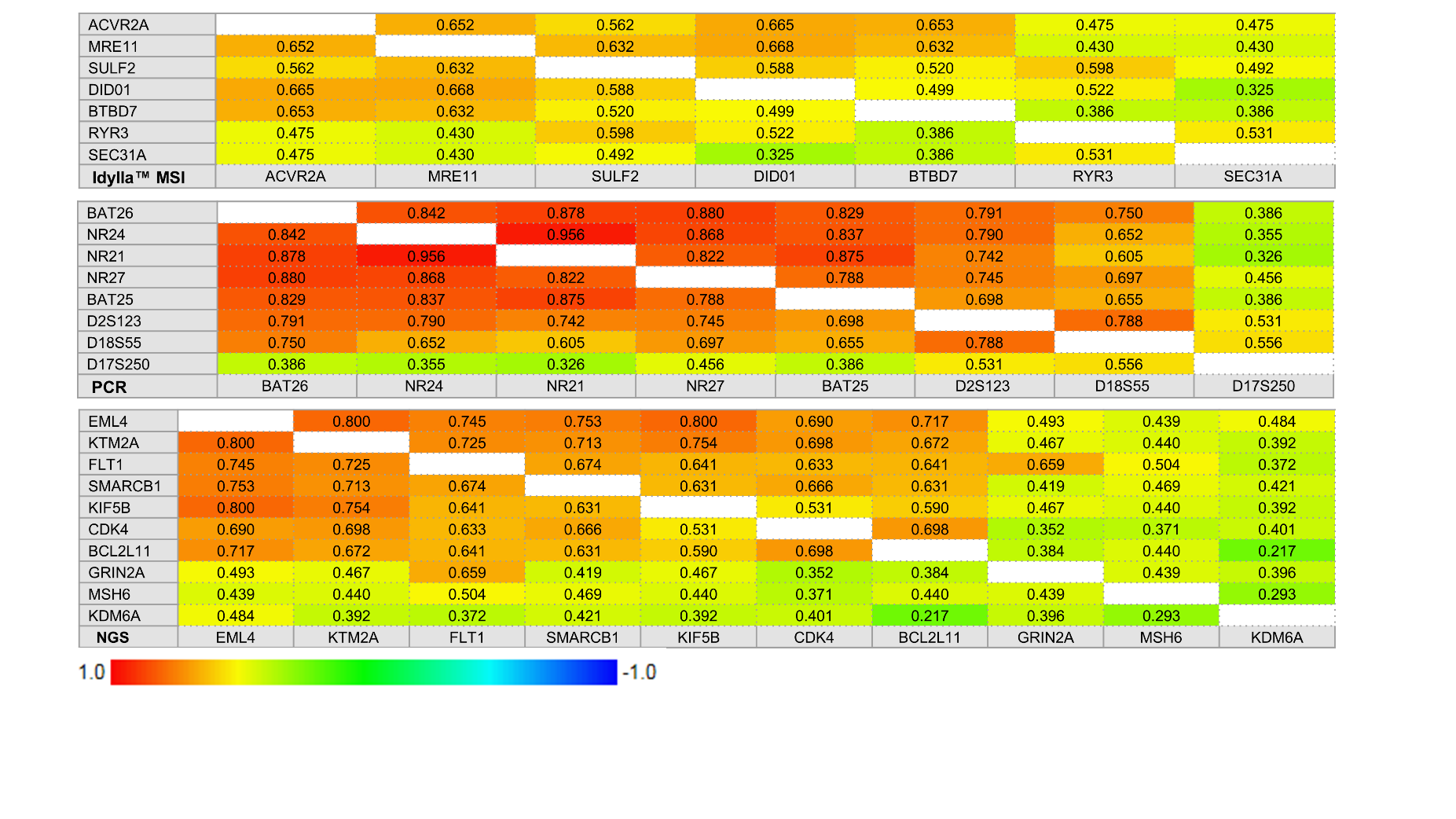


**References**

1 Salipante, S. J., Scroggins, S. M., Hampel, H. L., Turner, E. H. & Pritchard, C. C. Microsatellite instability detection by next generation sequencing. *Clin Chem* **60**, 1192-1199, doi:10.1373/clinchem.2014.223677 (2014).

2 Li, H. & Durbin, R. Fast and accurate long-read alignment with Burrows-Wheeler transform. *Bioinformatics* **26**, 589-595, doi:10.1093/bioinformatics/btp698 (2010).

3 Li, H. *et al.* The Sequence Alignment/Map format and SAMtools. *Bioinformatics* **25**, 2078-2079, doi:10.1093/bioinformatics/btp352 (2009).

4 Koboldt, D. C. *et al.* VarScan 2: somatic mutation and copy number alteration discovery in cancer by exome sequencing. *Genome Res* **22**, 568-576, doi:10.1101/gr.129684.111 (2012).

5 Boland, C. R. *et al.* A National Cancer Institute Workshop on Microsatellite Instability for cancer detection and familial predisposition: development of international criteria for the determination of microsatellite instability in colorectal cancer. *Cancer Res* **58**, 5248-5257 (1998).

6 Umar, A. *et al.* Revised Bethesda Guidelines for hereditary nonpolyposis colorectal cancer (Lynch syndrome) and microsatellite instability. *J Natl Cancer Inst* **96**, 261-268, doi:10.1093/jnci/djh034 (2004).

7 Suraweera, N. *et al.* Evaluation of tumor microsatellite instability using five quasimonomorphic mononucleotide repeats and pentaplex PCR. *Gastroenterology* **123**, 1804-1811, doi:10.1053/gast.2002.37070 (2002).

8 Nardon, E. *et al.* A multicenter study to validate the reproducibility of MSI testing with a panel of 5 quasimonomorphic mononucleotide repeats. *Diagn Mol Pathol* **19**, 236-242, doi:10.1097/PDM.0b013e3181db67af (2010).

9 Goel, A., Nagasaka, T., Hamelin, R. & Boland, C. R. An optimized pentaplex PCR for detecting DNA mismatch repair-deficient colorectal cancers. *PloS one* **5**, e9393, doi:10.1371/journal.pone.0009393 (2010).

10 Zhao, H. *et al.* Mismatch repair deficiency endows tumors with a unique mutation signature and sensitivity to DNA double-strand breaks. *Elife* **3**, e02725, doi:10.7554/eLife.02725 (2014).
